# Supplementary material for: Gut Reactions: How Far Are We from Understanding and Manipulating the Microbiota Complexity and the Interaction with Its Host? Lessons from Autism Spectrum Disorder Studies
Source: Nutrients. 2021 Oct 2;13(10):3492. doi: 10.3390/nu13103492 (PMC8538077; doi:10.3390/nu13103492)
Supplement: Supplementary file 1 [file nutrients-13-03492-s001.zip › nutrients-1394634-supplementary.pdf]

Table S1. studies investigating gut microbiome composition in ASD subjects.

Ref	Country	Subjects	Age (years)	Main outcomes (ASD compared to controls)	Samples	Microbiology assessment	Correlation with GI symptoms
Strati et al. (1) 2017	Italy	ASD:40<br><br>NT:40	11.1 ± 6.8<br><br>9.2 ± 7.9	**Phylum**<br>*Bacteroidetes*↓<br><br>**Genus**<br>*Alistipes, Bilophila, Dialister, Prevotella, Parabacteroides*, and *Veillonella*↓<br>*Collinsella, Corynebacterium, Dorea*, and *Lactobacillus*↑<br>*Candida*↑	Stool samples	16S rRNA (bacterial V3–V5 hypervariable regions) and ITS sequencing (fungal ITS1 rDNA region)<br><br>Pyrosequence	High levels of bacterial taxa belonging to *Escherichia/Shigella* and *Clostridium cluster XVIII* in constipated autistic subjects.
Liu et al. (2) 2019	China	ASD: 30<br>NT: 20	4.43 ± 1.47<br><br>4.28 ± 1.00 years	**Phylum**<br>*Firmicutes*↓<br>*Acidobacteria*↑<br><br>**Family**<br>*Veillonellaceae* and *Enterobacteriaceae*↑<br>*Ruminococcaceae, Streptococcaceae, Peptostreptococcaceae* and *Erysipelotrichaceae*↓	Stool samples	16S rRNA<br>(V3-V4 hypervariable regions)<br>MiSeq Illumina platform	Enriched *Fusobacterium, Barnesiella, Coprobacter* and reduced butyrate-producing taxa in constipated autistic subjects.
Iovene et al. (3) 2017	Italy	ASD: 47<br>NT: 33	6.0 ± 2.8<br><br>7.3± 3.1 years	**Species**<br>*Lactobacillus spp.* ↓<br>*Clostridium spp.* ↓<br>*Candida spp.* ↑	Stool samples	Culture	Constipation and alternating bowel did correlate with an increased permeability to lactulose, probably due to the reduction of *Lactobacilli*.
Zhang et al. (4) 2018	China	ASD: 35<br>NT: 6	4.9±1.5<br><br>4.6±1.1 years	**Phylum**<br>*Bacteroidetes* ↑<br><br>**Genus**	Stool samples	16S rRNA<br>(V3-V4 regions)<br>Illumina HiSeq. 2500 platform	-

				Sutterella, Odoribacter and Butyricimonas ↑<br>*Veillonella* and *Streptococcus* ↓			
Ma et al.<br>(5)<br>2019	China	ASD: 45<br><br>NT: 45	7.04±1.19<br><br>7.27±1.07<br>years	**Family**<br>*Acidaminococcaceae* ↓<br><br>**Genus**<br>*Lachnoclostridium, Tyzzerella subgroup 4, Flavonifractor* and *unidentified Lachnospiraceae* ↓<br><br>**Species**<br>*Clostridium clostridioforme* ↑	Stool samples	16S rRNA<br>(V3-V4 regions)<br>IlluminaHiSeq2500 platform	-
Coretti et al.<br>(6)<br>2018	Italy	ASD: 11<br><br>NT: 14	35±5.7<br><br>35±8.4<br>months	**Phylum**<br>*Proteobacteria* and *Bacteroidetes* ↑<br>*Actinobacteria* ↓<br><br>**Family**<br>*Actinomycetaceae, Coriobacteriaceae, Bifidobacteriaceae, Gemellaceae* and *Streptococcaceae* ↓<br><br>**Species**<br>*Bifidobacterium longum*↓<br>*Faecalibacterium prausnitzii* ↑	Stool samples	16S rRNA<br>(V3-V4 regions)<br>Illumina Miseq system	-
Kang et al.<br>(7)<br>2018	USA	ASD: 21<br><br>NT: 23	10.1±4.1<br><br>8.4±3.4<br>years	**Genus**<br>*Prevotella, Faecalibacterium, Coprococcus* and *Haemophilus* ↓<br><br>**Species**<br>*Prevotella copri, Faecalibacterium prausnitzii* and *Haemophilus parainfluenzae* ↓	Stool samples	16S rRNA<br>(V2-V3 regions)<br>Genome Sequencer FLX Titanium System	-
Zou et al.	China	ASD: 48	2-7 years	**Phylum**	Stool samples	16S rRNA<br>Illumina MiSeq platform	-

(8)<br>2020		NT: 48	48 months	*Firmicutes*, *Proteobacteria*, and *Verrucomicrobia*↓<br>*Bacteroidetes*↑<br><br>**Genus**<br>*Bacteroides*, *Prevotella*, *Lachnospiracea_incertae_sedis*, and *Megamonas* ↑<br>*Clostridium XlVa*, *Eisenbergiella*, *Clostridium IV*, *Flavonifractor*, *Escherichia/Shigella*, *Haemophilus*, *Akkermansia*, and *Dialister*↓<br><br>**Species**<br>*Bacteroides vulgatus* and *Prevotella copri* ↑<br>*Bacteroides fragilis* and *Akkermansia muciniphila*↓			
Plaza-Dìaz et al.<br>(9)<br>2019	Spain	ASD: 48<br><br>NT: 57	2-6 years	**Phylum**<br>*Actinobacteria* and *Proteobacteria* ↑<br><br>**Class**<br>*Actinobacteria*, *Bacilli*, *Erysipelotrichi*, and *Gammaproteobacteria*↑<br><br>**Family**<br>*Bacillaceae*, *Bifidobacteriaceae*, *Corynebacteriaceae*, *Desulfohalobiaceae*, *Enterobacteriaceae*, *Enterococcaceae*, *Erysipelotrichaceae*, *Fusobacteriaceae*, *Microbacteriaceae*, and *Thermoactinomycetaceae* ↑<br>Lachnospiraceae ↓<br><br>**Genus**	Stool samples	16S rRNA<br>(V3 and V4 regions)<br>Miseq Illumina sequencing system	-

				Bacillus, Bifidobacterium, Butyrivibrio, Enterococcus, Hespellia, Prevotella↑<br><br>**Species**<br>*Clostridium bolteae*, and *Clostridium difficile* ↑			
Wang et al. (10) 2019	China	ASD: 43<br><br>NT : 31	4.51±2.23<br><br>3.14±1.73 years	**Phylum**<br>*Actinobacteria* ↑<br>**Species**<br>*Clostridium botulinum* and *Eggerthella lenta* ↑<br>*Bacteroides vulgatus* ↓	Stool samples	Shotgun metagenomic sequencing<br><br>Illumina Hiseq4000 sequencer	-
Dan et al. (11) 2020	China	ASD: 143<br><br>NT: 143	5.189±0.170<br><br>4.937 ±0.155 years	**Phylum**<br>*Firmicutes,*<br>*Proteobacteria* and *Actinobacteria* ↑<br>*Bacteroidetes*↓<br><br>**Genus**<br>*Dialister, Escherichia-Shigella,*<br>and *Bifidobacterium*↑<br>*Prevotella, Megamonas,*<br>and *Ruminococcus* ↓	Stool samples	16S rRNA sequencing<br>Illumina Hiseq platform	Constipation in ASD was correlated to an increased α-diversity. *Coprobacter, Barnesiella,* and *Veillonella* were increased in constipated ASD children, whereas *Fusobacterium* was reduced.
Ding et al. (12) 2020	China	ASD: 77<br><br>NT: 50	38.5 ± 11.7<br><br>42.9 ± 14.5 months	**Genus**<br>Unidentified *Clostridiales*, unidentified *Lachnospiraceae*, unidentified *Erysipelotrichaceae, Dorea, Collinsella,* and *Lachnoclostridium*↑<br>*Bacteroides, Faecalibacterium, Parasutterella*, and *Paraprevotella*↓	Stool samples	16S rRNA<br>(V4 region)<br>Illumina HiSeq platform	-
Pulikkan et al. (13)	India	ASD: 30<br><br>NT: 24	9.5	**Phylum**<br>*Firmicutes*↑	Stool samples	16S rRNA<br>(V3 region)<br>NextSeq500	-

2018			9.5 years	**Family**<br>*Lactobacillaceae, Bifidobacteraceae, Veillonellaceae*↑<br>*Prevotellaceae*↓<br><br>**Genus**<br>*Bifidobacterium, Lactobacillus, Megasphaera,* and *Mitsuokella*↑<br>Prevotella, *Faecalibacterium, Roseburia* ↓			
Cao et al. (14) 2021	China	ASD: 45<br><br>NT: 41	6.80±3.79<br><br>5.16±0.99 years	**Family**<br>Lachnospiraceae↓<br><br>**Genus**<br>*Bacteroides, Bifidobacterium*↓<br>*Clostridium, Desulfovibrio, Streptococcus, Neisseria, Bacillus, Proteus* and *Streptomyces* ↑	Stool samples	16S rRNA (V4 region) Illumina Hiseq apparatus	-
Ahmed et al. (15) 2020	Egypt	ASD: 41<br><br>NT sibling-group: 45<br><br>NT Unrelated-group: 45		**Genus**<br>ASD and their siblings compared to the NT unrelated-group:<br>*Bacteroides, Ruminococcus* ↑<br><br>ASD compared to their siblings:<br>*Bifidobacterium* ↑	Stool samples	SYBR Green Real-Time PCR	-
Alshammari et al. (16) 2020	Saudi Arabia	ASD: 57<br><br>NT: 57	3-12 years	**Species**<br>*Clostridium Perfringens* ↑	Stool samples	Culture	High levels of *C. perfringens* and its toxin gene (Cpb2) have been related to GI symptoms in ASD.
Khalil et al.	Egypt	ASD: 58	5.41±1.55	**Species**	Stool samples	Quantitative real-time PCR	No statistically significant correlation was found.

(17)<br>2021		NT sibling-group: 45<br><br>NT Unrelated-group: 45	4.31±3.23<br><br>5.36±2.6 years	*Clostridium difficile* is not specifically prevalent in ASD subjects.			
Zou et al. (18)<br>2021	China	ASD: 29<br><br>NT: 31	2-6 years	**Genus**<br>*Saccharomyces* ↑<br>*Aspergillus*↓<br><br>**Species**<br>*Aspergillus versicolor* ↓<br>*Saccharomyces cerevisiae* ↑	Stool samples	ITS2 region sequencing<br>Illumina MiSeq platform	-
Finegold et al. (19)<br>2017	USA	ASD: 33<br>NT: 13	2-9 years	**Species**<br>*Clostridium Perfringens* ↑	Stool samples	Culture	High levels of beta2-toxin positive *C. Perfringens* have been found in autistic children with GI symptoms.
Luna et al. (20)<br>2017	USA	ASD: 14<br><br>NT with GI symptoms: 15<br><br>NT without GI	8.5<br><br>10.5<br><br>5.5 years	**Order**<br>*Clostridiales* ↑<br><br>**Genus**<br>Clostridium, Lachnoclostridium, Flavonifractor↑<br>Dorea, Blautia, Sutterella ↓<br><br>**Species**<br>*Clostridium lituseburense, Lachnoclostridium bolteae, Lachnoclostridium hathewayi,*	Rectal biopsy specimens	16S rRNA gene<br>(V1V3 and V4 regions)<br>MiSeq Illumina platform	ASD-GI subjects show a significant increase in several mucosa-associated *Clostridiales*.

		symptoms: 6		*Clostridium aldenense, Flavonifractor plautii* ↑<br>*Dorea formicigerans, Blautia luti, Sutterella spp.* ↓			
Kandeel et al. (21) 2020	Egypt	ASD: 30<br><br>NT: 30	4.4 ± 2.1 years	**Genus**<br>Clostridium ↑<br><br>**Species**<br>Clostridium paraputri, Clostridium bolteae, Clostridium diffiicile and Clostridium clostridiioforme ↑	Stool samples	Real-time PCR	-

References

1. Strati F, Cavalieri D, Albanese D, De Felice C, Donati C, Hayek J, et al. New evidences on the altered gut microbiota in autism spectrum disorders. Microbiome. dicembre 2017;5(1):24.
2. Liu S, Li E, Sun Z, Fu D, Duan G, Jiang M, et al. Altered gut microbiota and short chain fatty acids in Chinese children with autism spectrum disorder. Sci Rep. dicembre 2019;9(1):287.
3. Iovene MR, Bombace F, Maresca R, Sapone A, Iardino P, Picardi A, et al. Intestinal Dysbiosis and Yeast Isolation in Stool of Subjects with Autism Spectrum Disorders. Mycopathologia. aprile 2017;182(3–4):349–63.
4. Zhang M, Ma W, Zhang J, He Y, Wang J. Analysis of gut microbiota profiles and microbe-disease associations in children with autism spectrum disorders in China. Sci Rep. dicembre 2018;8(1):13981.
5. Ma B, Liang J, Dai M, Wang J, Luo J, Zhang Z, et al. Altered Gut Microbiota in Chinese Children With Autism Spectrum Disorders. Front Cell Infect Microbiol. 6 marzo 2019;9:40.
6. Coretti L, Paparo L, Riccio MP, Amato F, Cuomo M, Natale A, et al. Gut Microbiota Features in Young Children With Autism Spectrum Disorders. Front Microbiol. 19 dicembre 2018;9:3146.
7. Kang D-W, Ilhan ZE, Isern NG, Hoyt DW, Howsmon DP, Shaffer M, et al. Differences in fecal microbial metabolites and microbiota of children with autism spectrum disorders. Anaerobe. febbraio 2018;49:121–31.
8. Zou R, Xu F, Wang Y, Duan M, Guo M, Zhang Q, et al. Changes in the Gut Microbiota of Children with Autism Spectrum Disorder. Autism Res. settembre 2020;13(9):1614–25.
9. Plaza-Díaz J, Gómez-Fernández A, Chueca N, Torre-Aguilar M, Gil Á, Perez-Navero J, et al. Autism Spectrum Disorder (ASD) with and without Mental Regression is Associated with Changes in the Fecal Microbiota. Nutrients. 5 febbraio 2019;11(2):337.
10. Wang M, Wan J, Rong H, He F, Wang H, Zhou J, et al. Alterations in Gut Glutamate Metabolism Associated with Changes in Gut Microbiota Composition in Children with Autism Spectrum Disorder. Manichanh C, curatore. mSystems. 29 gennaio 2019;4(1):e00321-18.
11. Dan Z, Mao X, Liu Q, Guo M, Zhuang Y, Liu Z, et al. Altered gut microbial profile is associated with abnormal metabolism activity of Autism Spectrum

Disorder. Gut Microbes. 2 settembre 2020;11(5):1246–67.
12. Ding X, Xu Y, Zhang X, Zhang L, Duan G, Song C, et al. Gut microbiota changes in patients with autism spectrum disorders. J Psychiatr Res. ottobre 2020;129:149–59.
13. Pulikkan J, Maji A, Dhakan DB, Saxena R, Mohan B, Anto MM, et al. Gut Microbial Dysbiosis in Indian Children with Autism Spectrum Disorders. Microb Ecol. novembre 2018;76(4):1102–14.
14. Cao X, Liu K, Liu J, Liu Y-W, Xu L, Wang H, et al. Dysbiotic Gut Microbiota and Dysregulation of Cytokine Profile in Children and Teens With Autism Spectrum Disorder. Front Neurosci. 10 febbraio 2021;15:635925.
15. Ahmed SA, Elhefnawy AM, Azouz HG, Roshdy YS, Ashry MH, Ibrahim AE, et al. Study of the gut Microbiome Profile in Children with Autism Spectrum Disorder: a Single Tertiary Hospital Experience. J Mol Neurosci. giugno 2020;70(6):887–96.
16. Alshammari MK, AlKhulaifi MM, Al Farraj DA, Somily AM, Albarrag AM. Incidence of Clostridium perfringens and its toxin genes in the gut of children with autism spectrum disorder. Anaerobe. febbraio 2020;61:102114.
17. Khalil M, Azouz HG, Ahmed SA, Gad HA, Omar OM. Sensory Processing and Gastrointestinal Manifestations in Autism Spectrum Disorders: No Relation to Clostridium difficile. J Mol Neurosci. gennaio 2021;71(1):153–61.
18. Zou R, Wang Y, Duan M, Guo M, Zhang Q, Zheng H. Dysbiosis of Gut Fungal Microbiota in Children with Autism Spectrum Disorders. J Autism Dev Disord. gennaio 2021;51(1):267–75.
19. Finegold SM, Summanen PH, Downes J, Corbett K, Komoriya T. Detection of Clostridium perfringens toxin genes in the gut microbiota of autistic children. Anaerobe. giugno 2017;45:133–7.
20. Luna RA, Oezguen N, Balderas M, Venkatachalam A, Runge JK, Versalovic J, et al. Distinct Microbiome-Neuroimmune Signatures Correlate With Functional Abdominal Pain in Children With Autism Spectrum Disorder. Cell Mol Gastroenterol Hepatol. marzo 2017;3(2):218–30.
21. Kandeel WA, Meguid NA, Bjørklund G, Eid EM, Farid M, Mohamed SK, et al. Impact of Clostridium Bacteria in Children with Autism Spectrum Disorder and Their Anthropometric Measurements. J Mol Neurosci. giugno 2020;70(6):897–907.

Table S2. studies investigating the effects of gut microbial-based treatments in ASD patients and rodent models.

**Study/<br><br>Country**	**Study design**	**Subjects**	**Type of interventio n**	**Intervention details**	**Dose and frequency of intervention**	**Duration**	**Key findings**
Shaaban et al., 2017 [1] Egypt	A prospective, open-label study	30 ASD subjects (Age range: 5-9 years old)	Probiotics	Each gram contains 100x10^{6} CFU of three probiotic strains, including *Lactobacillus acidophilus, Lactobacillus rhamnosus* and *Bifidobacterium longum*	5g/day, administered once daily	3 months	Increased counts of Bifidobacteria and Lactobacilli; Improvements in both GI and core symptoms of ASD
Santocchi et al.,<br>2020 [2]<br>Italy	Double-blind randomized placebo-controlled trial	85 ASD children, 22 lost<br>(Age range: 18-72 months old)	Probiotics	**Intervention group (N=42):** Each packet contained 450 billions of eight probiotic strains,<br>including *Streptococcus thermophilus*, *Bifidobacterium breve*, *Bifidobacterium longum*, *Bifidobacterium infantis*, *Lactobacillus acidophilus*, *Lactobacillus plantarum*, *Lactobacillus para-casei, Lactobacillus delbrueckii subsp. bulgaricus*<br>**Placebo group (N=43):**<br>4.4 g of maltose and silicon dioxide	2 packets/day for 1 month and 1 packet/day for the following 5 months	6 months	No statistically significant difference in autism severity compared with placebo.<br>ASD children with GI symptoms experienced improvements in GI complaints, adaptive functioning, developmental pathways, and multisensory processing.<br>Autistic children without GI symptoms showed improvements in core symptoms of ASD
Arnold et al.2019 [3]<br>USA	Randomized, crossover pilot trial	13 ASD children, 3 lost<br>(Age range: 3-12 years old)	Probiotics	**Probiotic supplementation:** Each half packet contains 900 billion bacteria, including L. *casei, Lactobacillus plantarum, Lactobacillus acidophilus*, and *Lactobacillus delbrueckii* subsp. *Bulgaricus, B. longum,*	Half packet twice daily during the first 4 weeks; full packet twice daily if no effect was observed at	19 weeks	Suggested health benefit in children with ASD and GI complaints

				Bifidobacterium infantis, *Bifidobacterium breve*), *S. thermophiles*.<br><br>**Group1 (N=6) :** 8 week probiotic supplementation+3-week washout + 8 week placebo<br>**Group2 (N=4):** 8 week placebo +3-week washout + 8 probiotic supplementation	4 week and 15-week visit		
Wang et al. 2020 [4] China	Double-blind, placebo-controlled intervention study	26 ASD children (Age range: 2-8 years old)	Probiotics+ Prebiotics	**Intervention group (N=16):** Fructo-oligosaccharide+ *Bifidobacterium infantis*Bi-26, *Lactobacillus rhamnosus*HN001, *Bifidobacterium lactis*BL-04, and *Lactobacillus paracasei*LPC-37 (10^{10}CFU/pack/day)<br><br>**Placebo group (N=10):** maltodextrin	10^{10}CFU/pack/day	3.6 months	Significant reduction in GI symptoms and autism severity; fecal SCFAs, plasma neurotransmitters and related metabolites approached values similar to those in NT controls; decreased counts of *Clostridium*'s relative abundance and increased levels of *B. longum*
Tabouy et al. 2018 [5], Israel		Shank3 KO mice	Probiotics	Mice were gavaged 10^9 bacteria of *L. reuteri* reconstituted in a volume of 200 µl of PBS	Twice a week	3 weeks	Modifications in GABA receptor expression and protein levels in the brain; partial attenuation of unsocial behaviors in male mice and repetitive patterns in both male and female mice
Kang et al. 2017 [6], USA	An open-label clinical trial	18 ASD children (Age range: 7-16 years old)	MTT	Day 1-14: Vancomycin (40mg/kg per day, divided into three doses not to exceed 2 mg per day);		18 weeks	Increased bacterial diversity and increased counts of Bifidobacterium; improvements in both GI and core symptoms

				Day 12-74: Prilosec 20 mg daily<br>Day 15: MoviPrep standard kit with half dosage administered at 10 am and the other half at 4 pm<br>Day 16: participants received either oral administration of SHGM for 2 days (2.5×10^{12} cells/day divided into three oral doses) or a single rectal dose of SHGM (2.5×10^{12} cells/day).<br>Then, the first subgroup received lower oral doses for 8 weeks directly after the major initial dose (2.5×10^{9} cells) while participants who received the initial rectal dose waited for 1 week and then were administered with lower oral doses for 7 weeks (2.5×10^{9} cells)<br>+ 8-week follow up			of ASD; beneficial effects persisted after 8 weeks from the end of the treatment
Goo et al. 2020 [7], Korea		*Fmr1* KO mice	FMT	FMT from normal mice to *Fmr1* KO mice was conducted daily for 4 weeks		4 weeks	Improved autistic-like behaviors, especially memory deficits and social withdrawal; increased counts of *A. municiphila;* decreased brain levels of TNFα and Iba1

Chen et al. 2020 [8] China		MIA-induced ASD mice	GMT	The offspring were divided into four groups for oral gavage treatment: Control healthy mice treated with 200 μL saline; MIA-offspring treated with 200 μL saline; MIA-offspring treated with original donor gut microbiota suspended in 200 μL saline; MIA-offspring treated with cultured microbiota from pooled original human gut microbiota suspended in 200 μL saline	the treatment was implemented every other day 7 times.		Improved anxiety-like and stereotyped behaviors; improved serum levels of chemokines; improved gut microbiome composition
Grimaldi et al. 2018 [9] England	Randomized, double-blind, placebo-controlled trial	30, 4 lost (Age range: 4-11 years old)	Prebiotics	**Prebiotic:** B-GOS mixture (Bimuno® ; 1.8g: 80% GOS) **Placebo:** Maltodextrin-GLUCIDEX® 1.8 g	1.8g/day	6 weeks	Improved GI and core symptoms of ASD; increased counts of Bifidobacteria and Lachnospiraceae family; improved metabolic profiles
Liu et al., 2017[10] China	Single-blind, non randomized intervention pilot study	64 ASD children (Age range: 1-8 years old)	Vitamin A	Participants with an insufficient plasma retinol status (<1.05 μmol/L) received Vitamin A supplementation	Once with a dose of 200,000 IU	6 months	No significant differences in ASD-related symptoms; Increased proportion of *Bacteroidetes/Bacteroidales*; Decreased levels of *Bifidobacterium*
Newell et al., 2016 [11] Canada		BTBR mice	Ketogenic Diet		Mice were fed a KD for 10-14 days	7 weeks	Reduced total gut microbial counts in both cecal and fecal matter; increase in Firmicutes, especially *C. coccoides and C. leptum* and decrease in Bacteroidetes

Sanctuary et al., 2019 [12] USA	Randomized, double-blind, crossover clinical trial	20 ASD children, 12 lost (Age range:2-11 years old)	Bovine colostrum product+ probiotics	**Group1:** BCP+*B.fragilis* for 5 weeks+ 2-week washout+ BCP only for 5 weeks **Group2:** BCP only for 5 weeks+2-week washout+ BCP+*B.fragilis* for 5 weeks	Probiotics: 20 billion CFU/day BCP: 0.15g/Ib body weight per day	12 weeks	Improvements in both GI symptoms and aberrant behaviors were observed. Beneficial effects resulted to be more prominent in the BCP only group

References

1. Shaaban, S.Y.; El Gendy, Y.G.; Mehanna, N.S.; El-Senousy, W.M.; El-Feki, H.S.A.; Saad, K.; El-Asheer, O.M. The Role of Probiotics in Children with Autism Spectrum Disorder: A Prospective, Open-Label Study. *Nutr. Neurosci.* **2018**, *21*, 676–681, doi:10.1080/1028415X.2017.1347746.

2. Santocchi, E.; Guiducci, L.; Prosperi, M.; Calderoni, S.; Gaggini, M.; Apicella, F.; Tancredi, R.; Billeci, L.; Mastromarino, P.; Grossi, E.; et al. Effects of Probiotic Supplementation on Gastrointestinal, Sensory and Core Symptoms in Autism Spectrum Disorders: A Randomized Controlled Trial. *Front. Psychiatry* **2020**, *11*, 550593, doi:10.3389/fpsyt.2020.550593.

3. Arnold, L.E.; Luna, R.A.; Williams, K.; Chan, J.; Parker, R.A.; Wu, Q.; Hollway, J.A.; Jeffs, A.; Lu, F.; Coury, D.L.; et al. Probiotics for Gastrointestinal Symptoms and Quality of Life in Autism: A Placebo-Controlled Pilot Trial. *J. Child Adolesc. Psychopharmacol.* **2019**, *29*, 659–669, doi:10.1089/cap.2018.0156.

4. Wang, Y.; Li, N.; Yang, J.-J.; Zhao, D.-M.; Chen, B.; Zhang, G.-Q.; Chen, S.; Cao, R.-F.; Yu, H.; Zhao, C.-Y.; et al. Probiotics and Fructo-Oligosaccharide Intervention Modulate the Microbiota-Gut Brain Axis to Improve Autism Spectrum Reducing Also the Hyper-Serotonergic State and the Dopamine Metabolism Disorder. *Pharmacol. Res.* **2020**, *157*, 104784, doi:10.1016/j.phrs.2020.104784.

5. Tabouy, L.; Getselter, D.; Ziv, O.; Karpuj, M.; Tabouy, T.; Lukic, I.; Maayouf, R.; Werbner, N.; Ben-Amram, H.; Nuriel-Ohayon, M.; et al. Dysbiosis of Microbiome and Probiotic Treatment in a Genetic Model of Autism Spectrum Disorders. *Brain. Behav. Immun.* **2018**, *73*, 310–319, doi:10.1016/j.bbi.2018.05.015.

6. Kang, D.-W.; Adams, J.B.; Gregory, A.C.; Borody, T.; Chittick, L.; Fasano, A.; Khoruts, A.; Geis, E.; Maldonado, J.; McDonough-Means, S.; et al. Microbiota Transfer Therapy Alters Gut Ecosystem and Improves Gastrointestinal and Autism Symptoms: An Open-Label Study. *Microbiome* **2017**, *5*, 10, doi:10.1186/s40168-016-0225-7.

7. Goo, N.; Bae, H.J.; Park, K.; Kim, J.; Jeong, Y.; Cai, M.; Cho, K.; Jung, S.Y.; Kim, D.-H.; Ryu, J.H. The Effect of Fecal Microbiota Transplantation on Autistic-like Behaviors in Fmr1 KO Mice. *Life Sci.* **2020**, *262*, 118497, doi:10.1016/j.lfs.2020.118497.

8. Chen, K.; Fu, Y.; Wang, Y.; Liao, L.; Xu, H.; Zhang, A.; Zhang, J.; Fan, L.; Ren, J.; Fang, B. Therapeutic Effects of the In Vitro Cultured Human Gut Microbiota as Transplants on Altering Gut Microbiota and Improving Symptoms Associated with Autism Spectrum Disorder. *Microb. Ecol.* **2020**, *80*, 475–486, doi:10.1007/s00248-020-01494-w.

9. Grimaldi, R.; Gibson, G.R.; Vulevic, J.; Giallourou, N.; Castro-Mejía, J.L.; Hansen, L.H.; Leigh Gibson, E.; Nielsen, D.S.; Costabile, A. A Prebiotic Intervention Study in Children with Autism Spectrum Disorders (ASDs). *Microbiome* **2018**, *6*, 133, doi:10.1186/s40168-018-0523-3.

10. Liu, J.; Liu, X.; Xiong, X.-Q.; Yang, T.; Cui, T.; Hou, N.-L.; Lai, X.; Liu, S.; Guo, M.; Liang, X.-H.; et al. Effect of Vitamin A Supplementation on Gut Microbiota in Children with Autism Spectrum Disorders - a Pilot Study. *BMC Microbiol.* **2017**, *17*, 204, doi:10.1186/s12866-017-1096-1.

11. Newell, C.; Bomhof, M.R.; Reimer, R.A.; Hittel, D.S.; Rho, J.M.; Shearer, J. Ketogenic Diet Modifies the Gut Microbiota in a Murine Model of Autism Spectrum Disorder. *Mol. Autism* **2016**, *7*, 37, doi:10.1186/s13229-016-0099-3.

12. Sanctuary, M.R.; Kain, J.N.; Chen, S.Y.; Kalanetra, K.; Lemay, D.G.; Rose, D.R.; Yang, H.T.; Tancredi, D.J.; German, J.B.; Slupsky, C.M.; et al. Pilot Study of Probiotic/Colostrum Supplementation on Gut Function in Children with Autism and Gastrointestinal Symptoms. *PLOS ONE* **2019**, *14*, e0210064, doi:10.1371/journal.pone.0210064.
